# Perceived autonomy support in individuals with Parkinson’s disease requiring emergency care: a cross-sectional pilot study

**DOI:** 10.1186/s42466-024-00340-5

**Published:** 2024-08-15

**Authors:** Barend W. Florijn, Erik W. van Zwet, Ad. A. Kaptein, Anne A. van der Plas

**Affiliations:** 1 Department of Neurology, Leiden University Medical Center, Albinusdreef 2, 2333 ZA Leiden, The Netherlands; 2 Einthoven Laboratory for Vascular and Regenerative Medicine, Leiden University Medical Center, Albinusdreef 2, 2333 ZA Leiden, The Netherlands; 3 Department of Biomedical Data Sciences, Leiden University Medical Center, Albinusdreef 2, 2333 ZA Leiden, The Netherlands; 4 Department of Medical Psychology, Leiden University Medical Center, Albinusdreef 2, 2333 ZA Leiden, The Netherlands; 5 Department of Neurology, Alrijne Hospital, Simon Smitweg 1, 2353 GA Leiderdorp, The Netherlands

**Keywords:** Parkinson’s disease, Emergency care, Patient autonomy, Decision-making

## Abstract

**Background:**

Individuals with Parkinson’s disease (PD) report a diminished perceived functional autonomy as their condition progresses. For those seeking emergency care, it is unknown whether the patient-physician relationship is instrumental in respecting patient autonomy. This study evaluated patient autonomy ideals in individuals with PD requiring emergency care and the perceived support for autonomy from emergency department physicians.

**Method:**

Individuals with PD (n = 36, average age 78.1 years) were surveyed using the Ideal Patient Autonomy questionnaire (IPA) and the Health Care Climate Questionnaire (HCCQ). A multivariable regression analysis assessed whether patients’ Hoehn and Yahr stage and IPA questionnaire results predicted HCCQ items.

**Results:**

The IPA questionnaire revealed that individuals with PD in need of emergency care emphasize the significance of medical expertise (IPA ‘doctor should decide’ theme 0.71) in decision-making and their desire to be fully informed about all potential risks (IPA ‘obligatory risk information’ theme 0.71). The average HCCQ values showed a decreasing trend across Hoehn and Yahr stages 1 to 5: 6.19, 6.03, 5.83, 5.80, and 5.23, respectively. HY scale values also influenced HCCQ items related to the physician’s role.

**Conclusion:**

In our cohort, individuals with Parkinson’s disease tend to rely on medical expertise for decision-making and prioritize complete risk information during emergency care. However, this autonomy support diminishes as functional disability levels increase.

**Supplementary Information:**

The online version contains supplementary material available at 10.1186/s42466-024-00340-5.

## Introduction

Parkinson’s disease (PD) is the fastest expanding neurological disease globally [1, 2] and characterized by a range of motor and non-motor symptoms with significant variation among individuals [3]. Because cognitive decline impacts about 90% of individuals with PD [4], disease progression could result in a reduced perceived functional autonomy [5, 6]. This impacts health-related quality of life (QOL) [7] which could influence the decision to initiate treatment and treatment adherence [8]. Therefore, optimizing self-management for individuals with PD to improve their participation in decision-making is crucial [3].

Understanding the preferences of individuals with PD in emergency care requires a robust patient-physician relationship to quickly recognize and address critical situations [9]. This relationship could support patient autonomy in decision-making, as evidenced by a study involving elderly patients seeking emergency care [10]. However, it is unclear how individuals with Parkinson’s disease, in need of emergency care, perceive the patient-physician relationship in the emergency care setting, and to what degree this relationship is viewed as supportive of autonomy in decision-making.

We hypothesized that the ideals of patient autonomy are influenced by Parkinson’s disease, impacting perceived autonomy support in emergency care. To assess a patient’s ideal level of autonomy during discussions with their physician in general, we used the Ideal Patient Autonomy questionnaire (IPA), a 14-item instrument designed to study various concepts of the principle of autonomy [11]. Subsequently, we investigated whether individuals with PD perceived their emergency department physician as autonomy supportive using the Health Care Climate Questionnaire (HCCQ) [12]. The findings from both surveys were examined and categorized based on the Hoehn and Yahr scale (a 5-point tool for assessing the progression of motor symptoms in Parkinson’s disease; HY-scale) to explore the relationship between perceived autonomy and PD-related disability.

## Methods

### Study design

This is a cross-sectional (descriptive) pilot study that primarily focuses on descriptive statistics. We used a minimum sample requirement of 30 given the reasonable minimum recommendation for a pilot study in survey research by Johanson and Brooks [13].

### Patient selection and data collection

Individuals diagnosed with PD based on the Movement Disorder Society Clinical Diagnostic Criteria [14] were considered eligible when visiting the emergency department (ED) of the Alrijne Hospital between January and August 2022. Exclusion criteria were dementia (as determined by using the Montreal Cognitive Assessment (MOCA) combined with an assessment for anxiety and depression using the Hospital Anxiety and Depression scale (HADS) and a neuropsychological examination), a hospital admission for an elective procedure or conditions that mimic Parkinson’s disease. Following patients’ discharge from the ED to their home environment, telephone surveys were conducted within a 4–6-week timeframe to administer the IPA and HCCQ questionnaires. Both the IPA and HCCQ scale were answered in Dutch (given that both the IPA scale [11] and the HCCQ scale [15] were validated in Dutch. The patients were not assisted in answering the scales.

### Data analysis of the individual patient autonomy (*IPA*) scale

The IPA is a 14-item normative questionnaire based on six moral concepts [11]. These concepts reflect different models of patient autonomy in decision-making. In this study we adhere to the implementation method previously outlined in a study examining the facilitators and barriers perceived by patients as significant for the adoption of shared decision-making in vascular surgery [16]. Therefore, we included five items in the IPA questionnaire (items 2, 4, 5, 8, and 10) that investigate whether the doctor should make decisions or is most knowledgeable. Five other IPA statements center on the patient who should decide (items 1, 6, 9, 11, and 14) while five other IPA items emphasize the right not to participate in decision-making (items 2, 4, 8, 10 and 12). Lastly, we included IPA item 2, 3, 7, 9 and 11 to assess the IPA ‘obligatory risk information’ theme (defined as the probability of encountering potential harm or negative consequences). Responses to these questionnaire items were scored on a two-dimensional scale (0 = negative answer, 1 = positive answer). The scores were summed, and a final average score was calculated (Table 2).

### Data analysis of the health care climate questionnaire (HCCQ)

The HCCQ comprises 15 items on a 7-point scale (1 = completely untrue, 7 = completely true) and is designed to assess the degree to which individuals perceive their physician as autonomy supportive [17]. The scale score was computed by summing individual scores, and a final average score was calculated. Elevated average scores correspond to increased levels of autonomy support.

### Data analysis according to the Hoehn and Yahr scale (HY-scale)

We used the HY-scale to classify patients according to the progression of PD symptoms for organizing IPA and HCCQ average scores. The HY scale spans from stage 1, indicating unilateral involvement with minimal or no functional disability, to stage 5, which involves confinement to bed or wheelchair unless aided [18]. The HY scoring process took place after a neurological examination (conducted by AAvdP), and it was complemented by the information supplied by the patients and their caregivers (as assessed by BWF).

### Statistical analysis

A multivariable regression analysis was used to assess whether patients’ Hoehn and Yahr stage and IPA questionnaire results predicted HCCQ items. Multivariable regression analysis was performed using SPSS Statistics for Macintosh version 28 (IBM Corp. Released 2021. IBM SPSS Statistics for Macintosh, Version 28.0. Armonk, NY: IBM Corp).

## Results

### Clinical characteristics of included patients

The average age of patients included in this study was 78.1 (57–90) years (Table 1). The two most prevalent reasons for seeking emergency care were a systemic infection (n = 10) and a hip fracture (n = 6). Non-motor symptoms (such as impulsivity and depression) were not reported by all ED visiting patients in this study. All HY stages could be identified in the included patients, with stage 3 containing the largest number of patients (n = 11) and stage 5 the smallest number of patients (n = 2). Out of 82 individuals with PD who visited the ED, 36 patients independently completed both the IPA and HCCQ questionnaires (Table 1). 46 patients were excluded from participation due to various reasons: refusal to participate (n = 7), mild cognitive impairment (n = 8), or hospital admission for a severe medical condition (n = 23).

**Table 1:** Clinical characteristics of included patients (n = 36)

Sex, female, n (%)	18 (50%)
Age in years, average	78.1 (57–90)
Hoehn and Yahr stage	1 (n = 8)
	2 (n = 6)
	3 (n = 9)
	4 (n = 11)
	5 (n = 2)
Emergency Medicine diagnosis (n)	
Infection	10
Hip fracture	6
Heart failure	4
Parkinson’s disease progression	3
Head trauma	4
Cerebral infarction	2
Chest pain	2
Other	4

### Ideals of patient autonomy using the *IPA* questionnaire

Average IPA (SD) scores for the ‘patient should decide’ theme, ‘the right not to participate’ theme, the ‘doctor should decide’ and the ‘obligatory risk information’ theme were 0.67, 0.65, 0.71 and 0.71 respectively (Table 2). Given that HY stages are mainly depending on mobility, we compared IPA scores between patients with and without mobility problems (HY stage 1–2 vs. 3–5). Patients with HY stage 1–2 favored the ‘doctor should decide’ theme (0.77 ± 0.24) whereas patients with HY stage 3–5 favored the ‘obligatory risk information’ theme (0.71 ± 0.15) (Table 2). With respect to individual HY stages, patients classified as HY stage 1 and 2 also favored the ‘doctor should decide’ IPA theme. (Supplemental Table 1).

**Table 2:** Results of the Ideal Patient Autonomy questionnaire

Patient autonomy concept	Total (SD)	Hoehn and Yahr scale	
		1–2 (n = 14)	3–5 (n = 22)
‘Patient should decide’	0.67 (0.16)	0.63 (0.18)	0.69 (0.14)
‘Right not to participate’	0.65 (0.25)	0.71 (0.30)	0.62 (0.22)
‘Doctor should decide’	0.71 (0.20)	0.77 (0.24)	0.67 (0.17)
‘Obligatory risk information’	0.71 (0.15)	0.71 (0.18)	0.71 (0.15)

### Description of the health care climate questionnaire

The average value of the overall HCCQ was 5.88 ± 1.33. The lowest possible score in the total cohort of patients was found for HCCQ item number 4 with a score of 5.6 ± 1.46 (Table 3, HCCQ item number 13 is a negatively worded item). The HCCQ item number 8 exhibited elevated scores among patients with both HY stage 1–2 and HY stage 3–5, although its value was comparatively lower in the latter group (6.46 vs. 6.00). HCCQ scores and individual HY stages were inversely related with a decreasing average HCCQ value of 6.19, 6.03, 5.83, 5.80 and 5.23 found in patients with HY scores 1, 2, 3, 4 and 5 respectively (Supplemental Table 2).

**Table 3:** Description and results of the HCCQ-D items (text of the original English version)

HCCQ item	Total (SD)	Hoehn and Yahr scale	
		1–2 (n = 14)	3–5 (n = 22)
1. I feel that my physician has provided me choices and options	5.80 (1.39)	5.54 (1.81)	5.95 (1.09)
2. I feel understood by my physician	5.97 (1.34)	6.00 (1.08)	5.95 (1.50)
3. I can be open with my physician at our meetings	6.14 (1.35)	6.08 (1.19)	6.18 (1.47)
4. My physician conveys confidence in my ability to make changes	5.60 (1.46)	5.92 (1.26)	5.41 (1.56)
5. I feel that my physician accepts me	5.63 (1.37)	5.62 (1.19)	5.64 (1.50)
6. My physician has made sure I really understand about my condition and what I need to do	5.86 (1.31)	6.23 (0.93)	5.64 (1.47)
7. My physician encourages me to ask questions	5.69 (1.39)	6.00 (1.15)	5.50 (1.50)
8. I feel a lot of trust in my physician	6.17 (1.15)	6.46 (0.66)	6.00 (1.35)
9 My physician answers my questions fully and carefully	6.06 (1.03)	6.38 (0.65)	5.86 (1.17)
10. My physician listens to how I would like to do things	6.06 (0.84)	6.15 (0.80)	6.00 (0.87)
11. My physician handles people’s emotions very well	5.91 (1.27)	6.15 (0.99)	5.77 (1.41)
12. I feel that my physician cares about me as a person	6.03 (1.10)	6.08 (1.04)	6.00 (1.15)
13. I don’t feel very good about the way my physician talks to me	5.51 (2.02)	6.54 (0.66)	4.91 (2.31)
14. My physician tries to understand how I see things before suggesting a new way to do things	5.89 (1.02)	5.92 (0.95)	5.86 (1.08)
15. I feel able to share my feelings with my physician	5.91 (1.22)	6.23 (0.83)	5.73 (1.39)

### Multivariable regression to predict HCCQ-items

To assess the contribution of the physician in the HCCQ score, a multivariable regression was run to predict the HCCQ item 6 and 8 (item number specifically exploring the role of autonomy support by the physician) from sex, age, the HY stage and the average scores of the IPA questionnaire for each of the four models. These variables predicted the HCCQ item 6 score *F*(7,27) = 2.7, *p* < 0.05, R^2^ = 0.41, and the HCCQ item 8 score *F*(7,27) = 2.5, *p* < 0.05, R^2^ = 0.39 (Supplemental Tables 3 and 4).

## Discussion

This cross-sectional pilot study, involving 36 individuals with Parkinson’s Disease (PD) in need of emergency care, highlights their emphasis on the significance of medical expertise (IPA ‘doctor should decide’ theme 0.71) in decision-making and their desire to be fully informed about all potential risks (IPA ‘obligatory risk information’ theme 0.71). Moreover, individuals with PD expressed strong appreciation for the autonomy support provided by the treating physician in emergency care services. This was dependent on PD symptom progression as indicated by the results of the multivariable regression analysis, where HY scale values determined HCCQ items related to the physician’s role (item numbers 6 and 8).

Previous research has demonstrated that increased frailty correlates with a diminished perceived autonomy due to increased dependency and a decreased opportunity for decision-making [19]. In studies among patients with PD in primary care, general practitioners purposely chose a position that stimulates patients’ self-reliance and autonomy to underline their importance in decision-making [20]. In emergency care however, older people particularly value a state of autonomy that is exemplified by being well-informed and feeling in control, suggesting the importance of the patient-physician relationship that protects and promotes respect for patient autonomy [21]. When physicians endorse the autonomy of individuals with PD, it has the potential to strengthen the influence of personal control on both healthy behavior and psychological well-being. A comparable goal was achieved in a study that focused on promoting healthy behavior among 149 hypertensive patients with concurrent cardiovascular risk factors [22]. However, further research is required to validate this outcome in individuals with Parkinson’s disease, as the questionnaires utilized in our study do not explore the influence of personal control on healthy behavior.

With an average value of 5.88, the HCCQ total score in this cohort of PD patients displays a relatively high mean value. Previous studies using the HCCQ demonstrated that elevated HCCQ scores align with a higher quality of autonomous self-regulation, thereby enhancing overall quality of life [23]. Moreover, practical self-management support by physicians has shown to benefit health outcome [24]. This support is particularly achieved by an effective physician–patient relationship that empowers patients to manage their disease. In this study, the progression of PD symptoms, as indicated by HY grading, was associated with a reduced HCCQ total score. Since the HY classification is used to stage the functional disability associated with Parkinson’s disease, future studies are needed to elucidate the role of psychosocial symptoms related to PD. This could be of influence on management strategies, given that psychological adjustment significantly affects the health-related quality of life of individuals with Parkinson’s disease [25].

This study is subject to several limitations. First, only 36 out of the 82 included patients were able to participate and complete both questionnaires. Participants were recruited retrospectively after emergency department visits, with subsequent hospitalization in a sizable subset of patients (n = 23), which was an exclusion criterion for study participation. Secondly, there is a potential for recall bias in this cross-sectional study as the telephone surveys were conducted within a 4–6-week timeframe. However, we used carefully constructed questionnaires to obtain accurate and comprehensive information related to the specific topic of patient autonomy. Furthermore, although this is a cross-sectional study, the inclusion of a control group would sharpen our results significantly. For instance, including a control cohort with a different chronic (neurological) disease (e.g., multiple sclerosis) could identify alterations in thinking about autonomy specific to individuals with PD. In addition, it is important to consider other comorbidities in Parkinson’s disease, as the presence of additional health conditions can also impact autonomy. In addition, this study represents a single-centre pilot study investigation and independent replication is lacking. Therefore, an independent replication cohort is necessary to verify the results from this study. Furthermore, regarding the HCCQ results, physician performance may vary due to different emergency department personnel, fluctuating patient interaction time, daily patient volume changes, and severe emergency cases. Lastly, this study did not investigate how models of patient autonomy (IPA) or autonomy support (HCCQ) influence the decision for hospitalization of PD patients seeking emergency care. Therefore, future studies are needed to assess decision-making for hospitalization.

We conclude that in our cohort, individuals with Parkinson’s disease express a preference for a supportive autonomy approach when seeking emergency care, while autonomy support diminishes as functional disability increases. This aligns with the widely advocated patient-centered strategy in the care of individuals with Parkinson’s disease. Therefore, we suggest the following recommendations for clinical practice. First, emergency department physicians should be mindful of the motor symptoms of Parkinson’s disease that could impact the patient’s ability to express themselves. Second, autonomy support in emergency departments improves when physicians do not trivialize the way individuals with Parkinson’s disease articulate their symptoms.

### Supplementary Information

Supplementary Material 1.

## Data Availability

The datasets used and/or analysed during the current study are available from the corresponding author on reasonable request.
