# Supplementary material for: Perceived autonomy support in individuals with Parkinson’s disease requiring emergency care: a cross-sectional pilot study
Source: Neurol Res Pract. 2024 Aug 15;6:41. doi: 10.1186/s42466-024-00340-5 (PMC11325813; doi:10.1186/s42466-024-00340-5)
Supplement: Supplementary file 1 — Supplementary Material 1. [file 42466_2024_340_MOESM1_ESM.docx]

| Supplemental Table 1. Results of the Ideal Patient Autonomy questionnaire sorted by Hoehn and Yahr scale | | | | | |  |
| --- | --- | --- | --- | --- | --- | --- |
| Patient autonomy concept | Hoehn and Yahr scale | | | | | |
|  | 1  (n= 8) | 2  (n= 6) | 3  (n= 9) | 4  (n= 11) | 5  (n= 2) | |
| ‘Patient should decide’ | 0.56 (0.17) | 0.68 (0.18) | 0.73 (0.1) | 0.64 (0.17) | 0.80 (0) | |
| ‘Right not to participate’ | 0.68 (0.30) | 0.73 (0.32) | 0.62 (0.23) | 0.64 (0.23) | 0.50 (0.14) | |
| ‘Doctor should decide’ | 0.72 (0.23) | 0.80 (0.26) | 0.64 (0.19) | 0.69 (0.16) | 0.70 (0.14) | |
| ‘Obligatory risk information’ | 0.72 (0.18) | 0.70 (0.19) | 0.73 (0.14) | 0.69 (0.16) | 0.70 (0.14) | |

| Supplemental Table 2. Description and results of the HCCQ-D items | | | | | | |
| --- | --- | --- | --- | --- | --- | --- |
| HCCQ item | Total (SD) | Hoehn and Yahr scale | | | | |
|  |  | 1  (n= 8) | 2  (n= 6) | 3  (n= 9) | 4  (n= 11) | 5  (n= 2) |
| 1. I feel that my physician has provided me choices and options | 5.80 (1.39) | 5.8 (1.30) | 5.38 (2.13) | 5.78 (0.97) | 6  (1.26) | 6.5 (0.71) |
| 2. I feel understood by my physician | 5.97 (1.34) | 5.8 (1.30) | 6.13 (0.99) | 5.89 (1.05) | 6.09 (1.81) | 5.5 (2.12) |
| 3. I can be open with my physician at our meetings | 6.14 (1.35) | 6.2 (1.30) | 6  (1.20) | 6.67 (0.71) | 6.18 (1.08) | 4  (4.24) |
| 4. My physician conveys confidence in my ability to make changes | 5.60 (1.46) | 6  (1.22) | 5.88 (1.36) | 5.67 (1.00) | 5.27 (1.85) | 5.00 (2.83) |
| 5. I feel that my physician accepts me | 5.63 (1.37) | 5.6 (1.52) | 5.63 (1.06) | 6.22 (0.67) | 5.18 (1.83) | 5.5 (2.12) |
| 6. My physician has made sure I really understand about my condition and what I need to do | 5.86 (1.31) | 6.4 (0.89) | 6.13 (0.99) | 6.33 (0.50) | 5.36 (1.75) | 4.0  (1.41) |
| 7. My physician encourages me to ask questions | 5.69 (1.39) | 6.2 (0.84) | 5.88 (1.36) | 5.0 (1.00) | 6.0  (1.79) | 5.0  (1.41) |
| 8. I feel a lot of trust in my physician | 6.17 (1.15) | 6.8 (0.45) | 6.25 (0.71) | 5.89 (1.17) | 6.0  (1.61) | 6.5 (0.71) |
| 9 My physician answers my questions fully and carefully | 6.06 (1.03) | 6.8 (0.45) | 6.13 (0.64) | 6.0 (1.00) | 6.0  (1.26) | 4.5 (0.71) |
| 10. My physician listens to how I would like to do things | 6.06 (0.84) | 6.4 (0.55) | 6.00 (0.93) | 5.78 (0.97) | 6.27 (0.79) | 5.5 (0.71) |
| 11. My physician handles people’s emotions very well | 5.91 (1.27) | 6  (1.22) | 6.25 (0.89) | 5.33 (1.12) | 6.18 (1.54) | 5.5 (2.12) |
| 12. I feel that my physician cares about me as a person | 6.03 (1.10) | 5.8 (1.30) | 6.25 (0.89) | 5.89 (0.93) | 6.18 (1.25) | 5.5 (2.12) |
| 13. I don’t feel very good about the way my physician talks to me | 5.51 (2.02) | 6.6 (0.55) | 6.50 (0.76) | 5.44 (2.07) | 4.36 (2.58) | 5.5 (2.12) |
| 14. My physician tries to understand how I see things before suggesting a new way to do things | 5.89 (1.02) | 5.8 (1.30) | 6  (0.76) | 5.56 (1.13) | 6.27 (0.90) | 5.0 (1.41) |
| 15. I feel able to share my feelings with my physician | 5.91 (1.22) | 6.6 (0.55) | 6  (0.93) | 6  (1.00) | 5.64 (1.69) | 5.0 (1.41) |

Supplemental Table 3. Model summary and results of the multivariable regression to predict HCCQ-item 6.

| **Model Summary** | | | | |
| --- | --- | --- | --- | --- |
| Model | R | R Square | Adjusted R Square | Std. Error of the Estimate |
| 1 | .639^a^ | .408 | .255 | 1.130 |
| a. Predictors: sex, age, LI, PI, Hoehn Yahr scale, LL, SA | | | | |

| **ANOVA^a^** | | | | | | |
| --- | --- | --- | --- | --- | --- | --- |
| Model | | Sum of Squares | df | Mean Square | F | Sig. |
| 1 | Regression | 23.782 | 7 | 3.397 | 2.658 | .031^b^ |
|  | Residual | 34.504 | 27 | 1.278 |  |  |
|  | Total | 58.286 | 34 |  |  |  |
| a. Dependent Variable: Health Care Climate Questionnaire item 6 | | | | | | |
| b. Predictors: (Constant), sex, age, LI, PI, HY_scale, LL, SA | | | | | | |

| **Coefficients^a^** | | | | | | |
| --- | --- | --- | --- | --- | --- | --- |
| Model | | Unstandardized Coefficients | | Standardized Coefficients | t | Sig. |
|  |  | B | Std. Error | Beta |  |  |
| 1 | (Constant) | 8.334 | 2.385 |  | 3.495 | .002 |
|  | IPA_LL | 3.266 | 1.519 | .389 | 2.151 | .041 |
|  | IPA_LI | .089 | 1.257 | .011 | .071 | .944 |
|  | IPA_PI | -.640 | 1.831 | -.124 | -.350 | .729 |
|  | IPA_SA | .467 | 2.351 | .072 | .199 | .844 |
|  | HY_scale | -.267 | .184 | -.239 | -1.448 | .159 |
|  | age | -.055 | .031 | -.290 | -1.775 | .087 |
|  | sex | .732 | .405 | .284 | 1.806 | .082 |
| a. Dependent Variable: Health Care Climate Questionnaire item 6 | | | | | | |

Supplemental Table 4. Model summary and results of the multivariable regression to predict HCCQ-item 8.

| **Model Summary** | | | | |
| --- | --- | --- | --- | --- |
| Model | R | R Square | Adjusted R Square | Std. Error of the Estimate |
| 1 | .625^a^ | .391 | .233 | 1.007 |
| a. Predictors: (Constant), sex, Age, LI, PI, HY_scale, LL, SA | | | | |

| **ANOVA^a^** | | | | | | |
| --- | --- | --- | --- | --- | --- | --- |
| Model | | Sum of Squares | df | Mean Square | F | Sig. |
| 1 | Regression | 17.573 | 7 | 2.510 | 2.474 | .042^b^ |
|  | Residual | 27.399 | 27 | 1.015 |  |  |
|  | Total | 44.971 | 34 |  |  |  |
| a. Dependent Variable: Health Care Climate Questionnaire item 8 | | | | | | |
| b. Predictors: (Constant), sex, age, LI, PI, HY_scale, LL, SA | | | | | | |

| **Coefficients^a^** | | | | | | |
| --- | --- | --- | --- | --- | --- | --- |
| Model | | Unstandardized Coefficients | | Standardized Coefficients | t | Sig. |
|  |  | B | Std. Error | Beta |  |  |
| 1 | (Constant) | 6.184 | 2.125 |  | 2.910 | .007 |
|  | IPA_LL | 3.052 | 1.353 | .414 | 2.255 | .032 |
|  | IPA_LI | -1.590 | 1.120 | -.222 | -1.420 | .167 |
|  | IPA_PI | -5.486 | 1.631 | -1.210 | -3.363 | .002 |
|  | IPA_SA | 5.864 | 2.095 | 1.030 | 2.799 | .009 |
|  | HY_scale | -.161 | .164 | -.164 | -.980 | .336 |
|  | age | -.014 | .028 | -.083 | -.504 | .619 |
|  | sex | -.259 | .361 | -.114 | -.718 | .479 |
| a. Dependent Variable: Health Care Climate Questionnaire item 8 | | | | | | |
